# “A place to be safe, feel at home and get better”: including the experiential knowledge of potential users in the design of the first wet service in Montreal, Canada

**DOI:** 10.1186/s12954-022-00616-6

**Published:** 2022-04-06

**Authors:** Rossio Motta-Ochoa, Natalia Incio-Serra, Hélène Poliquin, Sue-Ann MacDonald, Christophe Huỳnh, Philippe-Benoit Côté, Jean-Sébastien Fallu, Jorge Flores-Aranda

**Affiliations:** 1 École de travail social, Université du Québec à Montréal, 455, boul. René-Lévesque Est Local W-4020, Montreal, Québec H2L 4Y2 Canada; 2 Faculty of Education, McGill University, 3700 McTavish Street, Montreal, Québec H3A 1Y2 Canada; 3 National Collaborating Center for Healthy Public Policies at Institut national de santé publique du Québec, 190 Boul Crémazie E, Montreal, Québec H2P 1E2 Canada; 4 École de travail social, Université de Montréal. Pavillon Lionel-Groulx, 3150, Jean-Brillant (C- 7069), Montreal, Québec H3T 1J7 Canada; 5 École de psychoéducation, Université de Montréal. Pavillon Marie-Victorin, 90, av. Vincent- d’Indy, Montreal, Québec H2V 2S9 Canada; 6 Département de psychiatrie et d’addictologie, Pavillon Roger-Gaudry, 2900, boul. Édouard- Montpetit, bureau S-750, Montreal, Québec H3T 1J4 Canada; 7 Institut universitaire sur les dépendances, 950 rue de Louvain Est, Montreal, Québec H2M 2E8 Canada; 8 Département de sexologie, Université du Québec à Montréal, 455, Boul. René-Lévesque Est, Montreal, Québec H2L 4Y2 Canada

## Abstract

**Background:**

The harmful use of alcohol is one of the leading health risk factors for people’s health worldwide, but some populations, like people who experience homelessness, are more vulnerable to its detrimental effects. In the past decades, harm reduction interventions that target these complex issues has been developed. For example, wet services include a wide range of arrangements (wet shelters, drop-in centers, transitory housing, etc.) that allow indoor alcohol use and Managed Alcohol Programs provide regulated doses of alcohol in addition to accommodation and services. Although the positive impacts of these interventions have been reported, little is known about how to integrate the knowledge of people experiencing homelessness and alcohol dependence into the design of such programs. The aim of this study is to present the findings of such an attempt in a first wet service in Montreal, Canada.

**Methods:**

Community based participatory research approach and qualitative methods—including semi-structured interviews and focus groups—were used to collect the knowledge of potential users (*n* = 34) of the wet service. The data collected was thematically analyzed.

**Results:**

Participants reported experiencing harsh living conditions, poverty, stigmatization and police harassment, which increased their alcohol use. The intersection between participants’ alcohol dependence and homelessness with the high barriers to access public services translated into their exclusion from several of such services. Participants envisioned Montreal’s wet service as a safe space to drink, a place that would provide multiple services, a home, and a site of recovery.

**Conclusions:**

Integrating the knowledge of potential users into the design of harm reduction interventions is essential to develop better and more adapted services to meet complex needs. We propose that it could fosters users’ engagement and contribute to their sense of empower, which is crucial for a group that is typically discriminated against and suffers from marginalization.

## Introduction

The harmful use of alcohol is a leading global risk factor for human health, and was the seventh cause of premature death and disability worldwide in 2016 [1]. Worldwide, around 3 million deaths and 132.6 million disability-adjusted years are attributable to alcohol consumption [2]. Harmful use of alcohol is also linked to noncommunicative diseases, being the attributable cause of 1.2 million deaths from digestive and cardiovascular disease, and 0.4 million deaths from cancer. Moreover, 107 million people are estimated to have alcohol use disorders worldwide [3]. Noticeably, harmful use of alcohol is not uniformly distributed. Some populations, such as people who experience homelessness, are more vulnerable to the detrimental effects of alcohol use [4, 5]. For example, the prevalence of alcohol dependence amongst people who experience homelessness in high income countries is around 38%, which is almost 10 times higher than among the general population [6].

Additionally, among persons who experience homelessness, there is a higher prevalence of numerous physical conditions directly related to alcohol dependence, including chronic inflammation of the digestive system, liver inflammation and cirrhosis, pancreatitis, hypertension, cardiomyopathy and coronary heart disease, alcohol-related seizures, and damage to the central nervous system [7, 8]. Moreover, people experiencing homelessness suffer from elevated rates of mental illness that often co-occur with alcohol use [9]. They are also more vulnerable to physical injures that result from falls, traffic accidents and assaults [10]. Furthermore, people who experience homelessness are exposed to the harsh living conditions of the streets (e.g., extreme weather, robbery, harassment, police profiling and discrimination), which contribute to the deterioration of their health [11]. Although it is reported that they overutilize emergency services for urgent medical care [12, 13], they also experience difficulties accessing primary and specialized health services, mental health services and housing [6, 14, 15]. Among the access barriers to public services of people experiencing homelessness, providers’ prejudices and judgemental attitudes toward them as well as their distrust of public services have been reported [16–18]. It is notable that the structural vulnerability^1^ of this population due to external forces such as poverty, violence and stigmatization as well as their alcohol dependence and limited access to public services negatively affects their health [20].

However, in the past decades, harm reduction interventions have been developed to address the specific health and social needs of persons who experience alcohol dependence and homelessness. For example, wet services (WSs) include a wide range of arrangements (e.g., wet shelters, drop-in centers,, transitory and permanent housing, elderly care facilities, etc.) offering a safe environment, support and services (e.g., food, activities, primary care, etc.) that allow indoor alcohol use [21]. Moreover, Managed Alcohol Programs (MAPs) that provide regulated doses of alcohol in addition to accommodation and services have proven to decrease use of non-beverage alcohol (e.g., hand sanitizer, mouth wash, rubbing alcohol, etc.), reduce alcohol-related harms, stabilize harmful drinking patterns, lessen emergency department visits, limit exposure to police contact, increase housing retention, foster recovery, in addition to improving social relations, wellbeing and quality of life [22–28]. Although there is a growing body of literature about the impacts of WSs and particularly MAPs on the health, living conditions and wellbeing of people who experience homelessness and alcohol dependence, still little is known about how these services are designed and how participation of potential users can be included in defining such services [29–31], which could contribute to better tailor them to these people’s needs.

To fill this gap in the literature, the present study aims to explore how a group of persons who experience alcohol dependence and homelessness use their experiential knowledge or the information and wisdom gained from lived experience [32] to envision Montreal’s first WS. We collected the participants’ perspectives to enrich the design of such service and better adapted it to their complex needs. In so doing, our objectives were: (1) to examine their experiences of alcohol use, homelessness, and use of or exclusion from public services, and (2) to explore the ways in which they envision a WS tailored to their specific needs and expectations.

## Approach and methods

This research is a component of a feasibility study to implement the first WS in Montreal, conducted by the Institut universitaire sur le dépendance (IUD). The study combines a community-based participatory research approach and qualitative methods to better grasp the experiential knowledge of potential users of the WS. Community-based participatory research fosters the participation of community members, people with lived experiences, organizational representatives, local authorities, and other stakeholders as co-researchers [33]. Further, it contributes to implementing effective interventions across diverse communities through strategies to redress power differences, foster mutual benefit among community and academic partners, and facilitate reciprocal knowledge translation [34]. For this study, IUD researchers worked closely with people who have lived experiences, representatives of community-based organizations that provide services and support to people experiencing homelessness, Indigenous organizations, health and social services, police services, the City of Montreal and a district where visible forms of homelessness are concentrated. A community advisory committee was made up of representatives from the above mentioned organizations. People with lived experience were involved in the community advisory committee through the organizations to which they were affiliated with. The community advisory committee participated in the study design, the development of research instruments and the recruitment of participants for the study. The community advisory committee also contributed to the data analysis by taking a critical look at the preliminary results. A total of 8 formal meetings were conducted with all members of the community advisory committee. Additionally, 5 informal meetings with people with lived experiences were conducted to obtain focused feedback on study design, research instruments, and analysis and dissemination of study results. Persons with lived experiences were compensated through the organizations to which they were affiliated.

Participants (*N* = 34) who had profiles similar to potential users of the WS were recruited. The eligibility criteria were (1) not having a fixed address over the last 12 months, (2) being engaged in heavy alcohol drinking^2^ and binge drinking^3^ over the last month, and self-identified as having alcohol dependence, (3) being aged 18 or older, (4) speaking French or English, and (5) having the capacity to understand and consent to participation. To ensure sample diversification [37] age, gender and ethnicity were also considered. Qualitative methods such as semi-structured interviews (*n* = 12) and focus groups (*n* = 2; with a total of 22 participants) were used to gather detailed descriptions of participants’ lived experiences [38]. Semi-structured interviews allowed researchers to thoroughly explore participants’ experiences of alcohol use, homelessness and use of or exclusion from services as well as how they envision a future WS. In the context of participatory research, community partners decided that certain topics that emerged in semi-structured interviews (e.g., gender differences that marked homeless experience, use of services, police harassment and profiling, etc.) that could affect WS design and implementation needed to be further explored. Participants with similar characteristics of those who participated in the semi-structured interviews were recruited for two focus groups. Focus groups also allowed eliciting collective views and disagreements among participants [39], revealing the diversity of experiences and expectations within this population. Semi-structured interviews lasting 20–80 min and focus groups lasting 75–100 min were recorded and digitally transcribed. Monetary compensation was offered to individuals who participated in semi-structured interviews and focus groups (CAN$40). Participants’ names were anonymized, and all additional identifying information was removed to protect participants’ confidentiality.

Audio transcripts of the semi-structured interviews and focus groups were thematically analyzed with the support of NVivo 12 software. Continous thematic analysis was conducted [40]. A mixed coding process (inductive and deductive) was completed and the collected data was reviewed through iterative analysis [41] to identify emergent themes and codes. The final thematic tree included 4 themes (clusters), 3 of which were chosen for this article (“Living conditions,” “Service Use,” and “Envisioning the WS”) and presented in the results section. These three themes included a total of 62 codes. To ensure validity, three researchers (RMO, NIS, JFA) analyzed over 10% of the gathered data, with 95% agreement [42]. To obtain consensus, researchers held face-to-face meetings during the study period. In keeping with the community-based participatory research approach of this study, preliminary analysis of the collected data was discussed in meetings with the community research partners, who provided their feedback. They contributed with their expertise to redefine the research process when needed.

## Results

In this section, we will examine participants’s structural vulnerability, homelessness and alcohol dependence; their use of services and experiences of exclusion; and their vision of an ideal WS.

### Structural vulnerability, homelessness and alcohol dependence

What follows explore how the socioeconomic factors as well as homelessness and alcohol use experience interact to define the participants’ structural vulnerability. The demographic information of the study participants is presented in Table 1. Of note, all participants had income of less than CAN$ 20,000 and a great majority had less than CAN$ 12,000, which is extremely low for Canadian standards of living.

**Table 1:** Demographic information

Characteristics	Mean or number
Mean age	49.4 years old; range, 24–71
Sex/gender	18 men; 13 women; 3 non-binaries
Nationality	32 Canadian; 2 non-Canadian
Province of birth	17 Quebec; 17 other Canadian provinces
First language	19 French; 15 English
Ethnicity	27 White; 7 Indigenous
Annual income	31 < 12,000; 3 = 12,001–20,000

All study participants identified as currently experiencing homelessness or as not having stable, safe, adequate and healthy homes [43]. Most participants indicated living in multiple places for various periods of time, including the streets, their own apartments or rooms, shelters, transitional houses, friends’ or family’s apartments, and hotel/motel rooms (see Table 2). All participants had lived on the streets during periods ranging from one month to fifteen years, and half of them reported living almost exclusively in the streets. Several had access to their own apartment or room through municipal and community programs, but participants noted their alcohol and drug use affected the ability to retain their housing. Sylvia, one of the participants, pointed out, “I often used my the rent money to pay my consumption, so I end up on the streets.” Other participants commented that certain practices associated with their substance use (e.g., having friends home, making too much noise, using common areas to consume substances) were sources of conflict with neighbors and landlords, and contributed to the loss of their housing.

**Table 2:** Places where participants lived

Condition	Number
Streets	34
Own apartment or room	12
Shelter	12
Transitional houses	7
Friends’ or family’s apartment	7
Hotel/motel room	6

All participants drank alcoholic beverages, and one fifth drank both alcoholic beverages and non-beverage alcohol (see Table 3). In addition to alcohol, the majority used other substances, the most common being cannabis, cocaine (cocaine powder and crack), amphetamines, prescription opioids and tranquilizers (benzodiazepines). Several participants defined themselves as “polydrug users,” who had to simultaneously managed “multiple addictions.”

**Table 3:** Participants’ alcohol and substance use

Patterns of alcohol use	Number
Participants who only use alcohol	7
Participants who use non-beverage alcohol in addition to alcohol and other substances	7
Participants who use alcohol and other substances	27

Substance(s) used in addition to alcohol	Number
Cannabis	2
Cocaine	3
Cannabis + cocaine	2
Cannabis + amphetamines	2
Cannabis + cocaine + amphetamines	2
Cannabis + cocaine + amphetamines + prescription opioids	2
Cannabis + cocaine + amphetamines + tranquilizers	3
Cannabis + cocaine + amphetamines + prescription opioids + tranquilizers, etc	11

The interaction between homelessness and alcohol/drug use contributed to shaping the participants’ structurally vulnerable position. While on the streets, participants reported experiencing harsh living conditions, instability and, consequently, widespread feelings of insecurity. Most noted that the extreme cold weather in Montreal caused frostbite, hypothermia and respiratory diseases, and increased their risks of death. They also reported being targets of frequent assaults and robberies. For example, Armand stated: “In the year and a half I’ve been on the streets, I’ve renewed seven, eight health insurance cards [because] my backpack got stolen eight times.” Many felt constantly stigmatized and judged by people in public areas because of their alcohol use and/or being homeless. Of note is the intense police harassment that the participants mentioned. Montreal Police has been publicly accused of social profiling,^4^ which is reflected by the participants’ descriptions. They reported that police often confiscated their alcohol or forced them to “pour it out”, as well as “tested” their sobriety with questionable means (e.g., “The cops asked me to walk in a straight line and I didn’t even have a car!”). Moreover, they reported that the police constantly handed out fines for drinking or being drunk in public areas, sleeping in subway stations and riding in the subway without paying. Many revealed that they accumulated “astronomical” debts due to their inability to pay the fines. They considered it a serious obstacle if they decided to getting off the streets and “reintegrate into society.” Thus, the harsh conditions of living on the streets and the associated stresses increased their substance use, as reported by Sandra: “You get drunk to stay warm and then you get vulnerable because you’re drunk and alone … and then you do drugs because you don’t wanna fall asleep … it’s just an endless cycle.”

### Use of services, public policies, and exclusion

Most participants considered that their substance use and/or experience of homelessness limited their access to several health and social services. For example, shelters in Montreal do not typically admit people under the influence of alcohol and other substances. According to Jack: “The problem is that when you’re a little drunk and you try to sleep in a shelter, [the staff] want to smell your mouth. [To get in], I have to eat a lot of gum and put on perfume!” In addition, most shelters forbid users to bring alcohol inside, which discouraged several participants from using them because of alcohol withdrawal (e.g., sweating, increased heart rate, hand tremors, agitation, seizures, etc.). Alcohol deprivation can severely compromise their health, as Mario stated: “I’m not currently eligible for shelters because in the morning I wake up and then I shake. If I don’t drink, I can end up in a hospital! It’s happened to me before, I had angina pectoris when I was without drink.” In addition, disease processes that accompany acute alcohol withdrawal can cause fits and even death [45]. Although all shelters have strict rules about forbidding alcohol use, some staff members had a more flexible approach and allowed participants to drink in their rooms to prevent health complications. As pointed out by Marco “In Blue Ceiling…they [the staff members] are nice…they just close their eyes and let you have your buzz in your room.”

Most were unemployed and their main source of income was social assistance (welfare.) However, many were often excluded from welfare support for short and/or long periods of time due to internal rules regarding leaving the province. Welfare benefits are easily suspended to anyone who leaves the province of Quebec for more than 15 cumulative days in a calendar month or more than 7 consecutive days in that month [46]. Moreover, as participants were frequently robbed, they did not always have identification cards to cash their welfare checks at a bank. In addition to welfare, almost all participants had street-based economic activities as complementary sources of income, such as panhandling, “squeegeeing” (wiping the windshields of cars stopped in traffic in exchange for money), sex work and petty drug trade. Due to drug-related petty crime and police tickets,^5^ several participants were incarcerated in provincial jails and federal prisons. For example, Cedric pointed out that “In the 1990s, I was put in jail for a year or so because of the tickets…I think that now this has changed, but at that time they put you in jail.” According to these individuals, their criminal records were among the factors that precluded them from being employed within the formal economy.

Participants had health issues commonly associated with alcohol use, including liver diseases, hypertension, and coronary heart disease (see Table 4). Several had chronic health conditions such as respiratory diseases and chronic pain that, as mentioned before, were provoked or exacerbated by harsh street life conditions. As residents of the province of Quebec, they were covered by the public health insurance plan to treat their health problems, but the fact that they frequently lost their identification cards limited their access to health services. They mostly used emergency departments and to do so, the police had to intervene. As Erica stated, “Lacking ID is hard, no health card, so the cops call the ambulance, and the [paramedics] have no choice to take you to the ER.” According to the research participants, only one clinic in Montreal (Médecins du monde) does not require an insurance card; but those who lived far from it and could not pay for public transportation were not willing to go there, as Gabrielle said: “I live in Vaudeville … I’m not gonna walk … all the way with my dog just to sit for a walk-in.” Moreover, some participants reported that “prejudices” associated with alcohol and drug use as well as with their homeless condition influenced how certain health providers treated them and discouraged them from seeking institutional health care. According to Max, “[The medical staff] look at you with these judgemental eyes … Stop looking at me like that! It’s not my fault I don’t have anywhere to live … I drink because I’m sick!” Additionally, some participants stated that they did not want to be hospitalized because most health providers did not consider alcoholism a “disease” and did not allow them to drink the alcohol they needed to prevent withdrawal symptoms.

**Table 4:** Participants’ physical and mental health conditions

Condition	Number
Liver disease	9
Hypertension	7
Coronary heart disease	7
Respiratory disease	5
Chronic pain	4
Diabetes	2
Arthritis	2
Hepatitis C	2
VIH	2
Depression	9
Anxiety disorder	8
Borderline personality disorder	6
Posttraumatic stress disorder	5
Schizophrenia	1

Several participants reported having one or more mental health conditions, the most frequent being depression, anxiety disorder and borderline personality disorder (see Table 4). Most of them received psychiatric treatment that was mostly pharmacological, the costs of which were fully covered by the Province of Quebec’s public drug insurance for people on social welfare [47]. However, some participants reported wanting psychotherapy and not being able to access it. Publicly funded psychological services are very limited [48] and are restricted to major mental health problems, as Mathieu reports: “I want psychological help … [but] it’s restricted … you have to be really sick … you have to be schizophrenic or something serious like that.”

Around one third of participants partook in various harm reduction programs for drug users provided by the government of Quebec through community-based organizations. They participated in needle and crack equipment distribution programs, supervised injection services, opioid agonist treatments, HIV and hepatitis C prevention and testing, etc. They considered that those programs were adapted to their needs and valued them, as Marie stated: “I appreciate Incognito, [a community-based organization that send] those buses, the night buses, where you can get tested for HIV, you can get condoms, latex-free condoms, and coffee.” Conversely to what they experienced in health services, they reported that the staff of community-based organizations had non-judgemental attitudes toward persons who use substances, which contributed toward creating environments in which they felt comfortable and welcomed. However, they highlighted that there were no equivalent harm reduction programs for alcohol users.

Until now, we have described how structural conditions such as homelessness, poverty, social stigmatization, and police harassment and profiling situate the participants in vulnerable positions, exacerbated by their alcohol dependence. Moreover, their structural vulnerability and alcohol dependence come into conflict with health and social services policies, limiting their access. During a focus group, while discussing their experiences of being rejected from shelters, Jeremey, Mathieu and Arthur described this situation as follows:

Jeremy: The minute [the staff] notice we’re intoxicated, they leave us outside, they don't want the trouble of taking care of us … they allow themselves to select those … who are less likely to cause problems.

Mathieu: They want the good homeless!

Arthur: But they should be helping those with problems …

Mathieu: Those who need more help!

By focusing on what the participants called “good homeless,” staff members of shelters and other services excluded those who were most vulnerable and in need of such services.

### Envisioning Montreal’s WS

We asked participants to envision an ideal WS. In doing so, we wanted to tap into their experiential knowledge to design a WS tailored to the specific needs and expectations of our target population. One participant depicted it as “a place to be safe, feel at home and get better,” which summarizes the expectations that most participants associated with this service. Below we describe in detail the main features that, according to the participants, a WS should have.

#### WS as safe space to drink

All participants considered that the WS should be a site where people could “consume alcohol safely.” Several participants would like the WS to operate like a “supervised injection site,” but for alcohol users. They highlighted their need for a “safe place” with “medical” staff available who could help them in case a “bad trip” or health complications arise while drinking.

Additionally, several participants suggested that it should be like a “drop-in centre” and a “shelter,” open “24/7,” where people can “drink alcohol indoors.” Marie noted that drinking alcohol on “the streets,” is “forbidden,” and thus “causes a lot of trouble” and exposes participants to more “risks.” Among the risks that the WS could prevent or reduce, participants listed “police harassment” and “tickets” for drinking or being intoxicated in public areas, being “assaulted” and “robbed” while drunk, as well as exposure to “cold weather” with consequences of “getting sick” and/or “dying in a snowbank.” Additionally, several participants envisioned using the WS to drink safely after waking up to prevent “withdrawal.” Moreover, some participants pointed out that the risks of living on the streets put them in a constant state of “alert and stress,” for which a place that gives them respite could bring a sense of security and thereby have a positive impact on their mental health.

To create this safe space, several participants considered that certain “flexible rules,” adapted to their lifestyles and needs were critical. For example, in contrast with most shelter policies, they wanted that WS users be able to keep their beds for “a week or so.” Also, most would like a rule that prevents users from getting “really, really drunk” in the WS because people who “drink too much” tend to become “violent,” which could be disruptive and threatening for others. In addition, several participants suggested that using drugs should not be an exclusion criterion to accessing WS, but that drug use inside should not be allowed. According to Oscar in relation to drug use: “there is already the injection site and we don’t want to bother people who don’t use drugs or have already quit.”

#### WS as place for the provision of multiple services

The limited access of the participants to health and social services strongly influenced how they envisioned an ideal WS. Many participants considered that the WS should offer temporary accommodation to alcohol users (wet shelter) who were regularly excluded from shelters and should facilitate their transition to stable forms of housing. Additionally, most considered that the WS should have medical staff not only to supervise their alcohol use, but to treat them for their chronic illnesses and transfer them to specialized medical services when needed. Furthermore, several participants highlighted that the WS should provide therapeutic services to address psychological problems they saw as closely related to their alcohol and drug use. According to Cedric, in the WS, “There should be workers like … psychologists because a lot of us have mental health problems and to treat them, we take our ‘medication’ … we drink … instead of [getting psychological] therapy.”

In addition, many participants would like to have at the WS a social worker to help them renew identification documents, to reapply for “welfare checks” when suspended, to get stable “housing,” to navigate their “problems with the legal system,” and to find a “job.” Moreover, several considered that the WS should provide occupational training to facilitate job “reintegration” and to be itself a source of employment. In one focus group, several participants exchanged ideas about this possibility and imagined together an entrepreneurial project suitable to their interests and needs, that would increase their sense of belonging and allow them to give back to the WS:

Mathieu: I think that [the WS] could be like a social reintegration program.

Cedric: We can make our own wine … I think it might be interesting to have a little project there for those who want to work … It would create jobs and then in addition you would have your drink!

Mathieu: Then, it could be a form of self-financing for the centre as well …

Max: I think …we should have responsibilities like cleaning …

Cedric: Volunteering, doing something, giving time … it creates a sense of belonging …

Oscar: Like in a monastery! [laughs]

Carl: Wine and cheese!

Cedric: A business run by homeless for … reintegrating into society.

Finally, almost all participants highlighted the need for workers to be well “qualified” in the provision of the multiple services as well as “well-paid” to prevent constant staff turnover. They also emphasized that they did not want workers to treat them like “children,” trying to “control” and “punish” them for breaking the rules. Instead, they would like workers to be “respectful,” non-judgemental, “compassionate,” “warm” and “caring.” In the case of outreach workers, some felt that they should have experiences of homelessness and/or consumption to be able to “understand” the users’ difficulties and challenges, and “adapt” the WS to users’ needs. As pointed out by Carl: “I don’t feel comfortable to share my problems with a 20-year-old who’s just finished university …it has to be someone like me, who drank in the past and understands what I’m talking about.”

#### WS as home

Several participants stated that, in addition to services, the WS should provide them with a sense of security and belonging that would make them feel “at home.” They considered that the WS could potentially be a suitable place to “remember” or “relearn” how to do some daily living activities not used when homeless such as “cooking,” “doing the laundry,” “cleaning,” and “making the bed.” Furthermore, they pointed out that living with others at the WS would allow them to improve their “social” and “communication” skills, thus consequently feel less isolated.

Noteably, several participants considered that the WS should help them to preserve their relationships with “significant others” such as partners, friends, family members and pets. In this sense, they would like to be able to have and/or host visitors at the WS. Moreover, they pointed out that the WS should be designed as a “pet-friendly” environment because several people experiencing homelessness have animals as companions. Most shelters forbid pets, which limited the participants’ access to such services, as Cedric described: “It would be super cool if [the WS] took animals! I spent two winters with my dog sleeping outside because none of the shelters take animals.”

Most participants considered that the WS should be designed as an inclusive environment where men, women, sexual minorities, couples, and diverse ethnic groups feel “at home.” However, some women who had experienced various forms of abuse perpetrated by men pointed out they preferred that the WS be segregated by sex/gender (“one WS for women and other for men”) or that the WS have private areas exclusively for women. In addition, some participants who identified as white would prefer a WS where there were no Indigenous people because in their words “Indigenous people dr[ink] too much” and/or do not “tolerate alcohol very well,” and thus they turn “violent” and “f[ight] with others.” Conversely, participants who identified as Indigenous considered that the WS should include people from different ethnicities and did not ask for a WS exclusively for Indidgenous people.

Several participants stated that they wanted the WS to be adapted to their cultural norms, preferences, practices and language. For example, white participants pointed out that they did not like the food many shelters provide because they felt it was “unfamiliar” and foreign. According to Renée, “[At the shelter] they gave us curry and 90% of us were Quebecers. We didn’t eat … I wonder why they don’t give us just sandwiches or something simple we like.” Alternatively, participants who identify as Indigenous indicated that at the WS they would like to have Indigenous games and activities and not only “Western” board games. Furthermore, some participants wanted bilingual (English and French) workers. According to participants from outside Quebec who only speak English, in most public services they attended providers did not speak English, which discouraged them coming back.

#### WS as site of recovery

Most participants considered that the WS should be a site of “recovery” from alcohol use and harsh living conditions. They stated that having a safe place to drink and live would “definitely” have a positive impact on their “physical and mental health,” as well as their general “wellbeing.” Moreover, building on their own perceptions of recovery, they would like the WS to offer therapeutic resources such as “detox,” “rehab,” “medication,” addiction “therapies,” and medical “follow up” for those who want to “stop,” “moderate” or reduce the harms associated to their drinking. Some suggested that the WS should host support groups such as Alcoholics Anonymous for those who want to stop drinking and stay sober.

Most participants commented that the WS should provide a form of “supervision” or “control” for those who want to moderate their drinking or reduce the associated harms. They consider that supervision should be “flexible enough” to allow users to drink without “feeling inhibited,” and to respect each person’s drinking pattern, but it had to prevent users from “excessive drinking” or binging. However, when we asked participants to imagine ways to monitor users’ drinking, their answers were vague as Carl’s response illustrates: “If there is no supervision [in the WS], it would be a mess! … but how to do it … for everyone … no idea.” When we explained that users could be given regulated doses of alcohol according to individualized plans (MAP), several believed that this “might work” for them. Others who considered they needed to drink “a lot” on every occasion were hesitant about the applicability of this type of monitoring. According to Marco, “I’m just a bad drunk and a wicked drunk … I can’t take just a drink because … I can’t stop!”.

Despite their hesitations, participants highlighted that being surrounded by people who try to stop drinking or to drink in a controlled way would be “very inspiring,” and when “ready” it would be a “motivation” to reduce their own drinking. Moreover, several participants suggested that to ensure users’ recovery, the WS should “redirect” their “attention to drinking” through artistic activities (“painting,” “writing,” “playing music”), film screenings, as well as games and sports. Some participants also considered that to “keep” users “busy” the WS should offer a variety of basic workshops on using the Internet and digital technologies.

Of note, a few participants disagreed with the possibility of implementing a WS because it would enable and/or increase users’ drinking, which would constitute an obstacle to their recovery. For example, Arleen considered that having a place that provides shelter and alcohol to users was “a terrible idea” that would “make it way too easy for homeless people,” who would “keep drinking.” Additionally, Sebastian pointed out that in a place where “everyone consumes, one would encourage the other” and the users would “end up” drinking more than when they were “alone.”

## Discussion

This article explores how a group of persons who experienced alcohol dependence and homelessness envisioned an ideal WS. Our study aims to contribute to the growing literature about WSs and MAPs that integrates the users’ perspectives to evaluate and improve harm reduction interventions for problematic alcohol use. These studies trace the effects of such interventions on users’ alcohol consumption, housing retention, encounters with police, emergency department visits, physical and mental health care and quality of life [22–28, 49, 50]. However, there are a few published descriptions on how to incorporate participants’ perspectives into the design phase of these services [29–31]. We consider that integrating the experiential knowledge of potential users in the design of interventions could help to develop WSs better adapted to users’ expectations and needs. Moreover, it could increase the possibilities of user engagement and thus the overall reach and impact of interventions and services as other studies on the field of health care science have shown [51–54]. Furthermore, involving users in the development of design is asserting the value of their knowledge and ability to actively participate in their own care, which might contribute to their empowerment.

Our findings aim to shed light on how excluding vulnerable populations from health and social services occurs. As some studies have shown, individuals with low incomes and who experience homelessness, violence, and/or exploitation are more likely to engage in heavy drinking, exhibit extreme intoxication and drink cheap and/or non-beverage alcohol [55, 56]. In addition, our study highlights that the intersection between participants’ structural vulnerability, alcohol dependence and health and social services regulations produced forms of exclusion despite the fact these services are actually citizenship rights. Social and health policies that regulate access to public services as well as the practices of service providers may be influenced by ideas of deservingness that distinguish between those who are worthy and unworthy of receiving support [57, 58]. This might be the case when staff members at shelters only allow access to what the participants called “good homeless” and left out those who are intoxicated and exhibit problematic behaviors, excluding those who need help the most. Moreover, as Bernie Pauly and colleagues have shown in a recent study about ethical tensions in public health systems, the ability of practitioners to disrupt stigma and discrimination is constrained by embedded patterns of exclusion that are part of the system itself [59]. Thus, despite being entitled to public services, structurally vulnerable individuals who are alcohol dependent repeatedly fall through the cracks of the health and social policy safety net.

Our study aims to contribute to the provision of inclusive services that attend to the needs of marginalized people who experience alcohol dependence and homelessness. Participants reported limited access and, to a lesser degree, refusal to attend health and social services, which is consistent with the literature about WSs and MAPs [49, 60–62]. This research also shows that users of WSs and MAPs find benefit in the various services and supports accessible at those facilities, such as housing and/or transition to stable housing, social services, primary care, addiction treatments, work, as well as food and meals [24, 27, 49, 50, 60, 63]. Along the same line, participants considered that Montreal’s WS should provide the multiple health and social services they did not receive in other care settings. Thus, co-locating multiple services (mental and physical health care, addiction treatment, and social assistance), often referred to as “one-stop shopping” [64], might be an optimal way to deliver comprehensive and integrated care at Montreal’s WS. However, in his study about a residential facility that combined health and social services with supervised drinking, Joshua Evans [65] warned about the paradox of inclusively caring for a population seen as undeserving of help in an enclosed location by excluding it from the rest of the city’s geography.^6^ Although initially, Montreal’s WS could provide multiple services in a single site, to thoroughly foster user inclusion, it is crucial to establish collaborations and coalitions with authorities and stakeholders toward implementing policies that lower the thresholds of existing public services in the long term. Additionally, interventions oriented towards reducing stigma and discrimination among health and social care providers could contribute to making public services more inclusive for persons experiencing alcohol dependence and homelessness [67].

This research also contributes to the growing literature pertaining to the importance of creating a sense of home that tailor WSs to the needs and expectations of people experiencing alcohol dependence and homelessness. Our findings in relation to the meanings that the participants attribute to home are consistent with previous studies that have shown that MAP users associate this notion with feelings of safety and belonging, sense of togetherness and connection with others, as well as with a space to enact activities of daily life and relearn social skills [6, 7]. Along with the findings of Bernnie Pauly and colleagues [8], our study participants also assigned cultural meanings to home, which they envisioned as a place where they can eat familiar food, play the games they like, and where others speak their language. Interestingly, when participants were asked to imagine an ideal WS, they began by depicting a dwelling place (e.g., a shelter, a dropping center, etc.) to which they associated practical functions (e.g., safety drinking, service provision, etc.). But as they continued envisioning the WS, they started expressing positive emotions and using the term “home” to describe such place. Although we need to further explore how persons experiencing alcohol dependence and homelessness conceptualize home, we consider that dentifying the specific meanings that they associate to this notion is crucial to operationalize it and to incorporate concrete elements and strategies into the design of the WS.

Our study results highlight the need to tailor services to the individual needs of people who experience alcohol dependence and homelessness. Far from being a uniform population, these people have a diverse range of experiences of alcohol use (e.g., those who consider that they drink a lot and binge frequently, and those who do not), substance use (e.g., those who use drugs in addition to alcohol and those who do not) and homelessness (e.g., those who mostly have unstable forms of housing and those who almost exclusively live in the streets). Their needs and expectations about WSs are also shaped by their gender (e.g., several women would like segregated services or spaces for females) and cultural norms. Interestingly, although all the participants agreed that the WS should be a site of recovery, they have diverse ways of conceiving their own recovery. Some envision their recovery within an abstinence framework (stop drinking), others within a harm reduction framework (reduce harms associated to drinking); some as a continuum between the two frameworks (first reducing harms associated to drinking, then progressively reducing drinking and eventually stopping it), requesting services that blur the distinctions between both frameworks. Further research about local notions of recovery among alcohol users is still needed, but our findings suggest that a person-centered approach would be more pertinent than a one-size-fits-all approach for Montreal’s WS to address the different needs and expectations of this population. Of note, implementing person-centered care into practice is highly demanding, particularly for health providers. For example, assessing each individual is labor intensive and requires a wide range of expertise [68]. However, research in the field of addictions has shown that applying the principles of person-centered care (e.g., individualized focus on care, holistic approach, shared decision-making, and enhanced therapeutic alliance between user and service provider, etc.) is associated with greater service use and user recovery [69–71]. Along this line, the provision of regulated doses of alcohol could constitute a privileged opportunity to apply such principles. Staff member and user could define together the user’s individualized drinking plan, check on her/his wellbeing and discuss additional harm reduction strategies adapted to her/his needs.

Certain limitations of our study should be highlighted. Due to its qualitative design, the participants’ sample does not intend to be representative of all people who experience alcohol dependence and homelessness. However, our detailed description of the participants’ experiences should help others appraise the transferability of our findings to similar populations. Additionally, our study is based on semi-structured interviews and focus groups; therefore, social desirability could have been induced. Nonetheless, the interviewers’ non-judgmental attitudes helped to control this potential bias. We could have used other qualitative techniques such as participant observation to enhance our study results. But since the goal was to explore participants’ experiences and expectations, we considered semi-structured interviews and focus groups to be the most appropriate methodological choice. Although for the participants’ recruitment, we used criteria that ensured sample diversification, the semi-structured interview and focus group guides did not include specific questions to deepen our exploration of differences by age, gender and ethnicity. Information about such differences included in this paper was mostly emergent. The perspective of Indigenous potential users of the Montreal’s WS needs to be further examinined.

## Conclusion

This study explored how a group of persons who experience alcohol dependence and homelessness used their experiential knowledge to envision the first WS in Montreal. Drawing on their homelessness and alcohol use experiences as well as on their service use and exclusion experiences, the participants imagined a WS as a safe space to drink, a place for the provision of multiple services, a home, and a site of recovery. Our findings aim to contribute to the growing literature about harm reduction interventions that address alcohol consumption by including the experiential knowledge of potential users into the design phase of these interventions. Our overall goal is that the experiential knowledge of these persons shed light on the development of interventions that respond to their needs and expectations, and thus ensure their engagement to such interventions. Finally, the study highlights the value of a community-based participatory research approach and qualitative methods (semi-structure interviews and focus groups) in capturing the lived experiences of participants.

## Data Availability

The database created and analyzed for this study is not publicly available. It is based on a small sample of research participants, which threatens their anonymity and confidentiality. It could be available from the corresponding author on reasonable request.

## Abbreviations

WS: Wet service
MAP: Managed Alcohol Program

## Footnotes

1 The notion of structural vulnerability as defined by James Quesada and colleagues refers to a positionality that imposes physical/emotional suffering on specific populations and individuals in patterned ways, which is the result of class, cultural, gender/sexual, and/or racialized discrimination [19]. In medical anthropology, this notion has been used to analyse groups of people that due to their subordinated location in society have higher rates of ill health and limited access to health resources.

2 To define heavy alcohol use, we used criteria of the National Institute on Alcohol Abuse and Alcoholism: (1) consuming more than 4 drinks on any day or more than 14 drinks per week for men or (2) consuming more than 3 drinks on any day or more than 7 drinks per week for women [35].

3 To define binge drinking, we use the criteria of the Substance Abuse and Mental Health Services Administration: (1) consuming 5 or more alcoholic drinks on the same occasion (i.e., at the same time or within a couple of hours of each other) on at least 1 day in the past month for males or (2) consuming 4 or more alcoholic drinks for females on the same occasion on at least 1 day in the past month for females [36].

4 Montreal Police’s social profiling practices have been the source of public debates and have been sparked by several research reports, which have called out these practices and have proposed specific recommendations to end them [44].

5 Until 2004, the municipal court of Montreal issued warrants of imprisonment for non-payment of tickets for minor infractions [44].

6 Evans calls this a paradoxical geography of inclusive exclusion. In another article [66], the first author has further discussed how interventions and practices that aim to foster social inclusion produce exclusions.
